# Surgical options for symptomatic old osteoporotic vertebral compression fractures: a retrospective study of 238 cases

**DOI:** 10.1186/s12893-020-01013-1

**Published:** 2021-01-06

**Authors:** Zhengwei Xu, Dingjun Hao, Liang Dong, Liang Yan, Baorong He

**Affiliations:** Department of Spine Sugery, Shanxi, Xi’an Jiaotong University Affiliated Honghui Hospital, No.555, Youyi Road, Beilin District, Xi’an, People’s Republic of China

**Keywords:** Symptomatic old osteoporotic vertebral compression fractures, Graded surgery, Visual analog score, Oswestry disability index, Sagittal index

## Abstract

**Background:**

Symptomatic osteoporotic vertebral compression fractures (OVCF) are increasing, as are acute and chronic pain episodes and progressive spinal deformities*.* However, there are no clear surgical treatment criteria for patients with these different symptoms. Therefore, this study aims to explore the surgical approaches for the treatment of OVCF with different symptoms and evaluate the feasibility of these surgical approaches.

**Methods:**

We retrospectively analyzed 238 symptomatic OVCF patients who entered our hospital from June 2013 to 2016. According to clinical characteristics and imaging examinations, these patients were divided into I-V grades and their corresponding surgical methods were developed. I, old vertebral fracture with no apparent instability, vertebral augmentation; II, old vertebral fracture with local instability, posterior reduction fusion internal fixation; III, old fractures with spinal stenosis, posterior decompression and reduction fusion and internal fixation; IV, old vertebral fracture with kyphosis, posterior osteotomy with internal fixation and fusion; V, a mixture of the above types, posterior osteotomy (decompression) with internal fixation and fusion. Postoperative visual analog score (VAS), oswestry disability index (ODI) scores, sagittal index (SI) and ASIA grades of neurological function were observed.

**Results:**

All 238 patients were followed up for 12–38 months, with an average follow-up of 18.5 months. After graded surgery, the VAS score, ODI score, and vertebral sagittal index SI of 238 patients were significantly improved, and the difference between the last follow-up results and the preoperative comparison was statistically significant (*P* ˂ 0.05). Besides, the postoperative ASIA grades of 16 patients with nerve injury were improved from 14 patients with preoperative grade C, 2 patients with grade D to 4 patients with postoperative grade D and 12 patients with postoperative grade E.

**Conclusion:**

In this study, we concluded that graded surgery could better treat symptomatic old OVCF and restore spinal stability. This provides clinical reference and guidance for the treatment of symptomatic old OVCF in the future.

## Background

As the population ages, osteoporotic vertebral compression fractures (OVCF) are increasing, as are acute and chronic pain episodes and progressive spinal deformities [1]. It has been reported that the prevalence of vertebral fracture in women over 50 years old is about 15%, and that in women over 80 years old is up to 36.6% [2]. As OVCF is a kind of low-energy damage, post-injury pain is easily confused with fatigue pain. Moreover, most elderly patients are not sensitive to pain, which may easily delay the disease and eventually develop into old fractures. For old OVCF, conservative treatment, such as bed rest, wearing braces, anti-osteoporosis and other drug treatment, has no obvious effect on most patients [3].

Vertebral compression fractures are the most common complication of spine osteoporosis, which can cause pain at the fracture site and loss of the vertebral body, and can lead to later kyphosis [4, 5]. The possible reason for the long-term pain at the fracture site is that the fractured vertebra is always in a state of continuous compression due to long-term activities, and the fractured vertebra is slightly displaced, which constantly stimulates the peripheral nerves of the vertebra. In addition, 14% of patients will develop pseudarthrosis [6]. Pseudarthrosis is also known as fracture nonunion, refers to the fracture end under the influence of certain conditions, fracture healing function stops, the fracture end has formed pseudarthrosis. In more severe cases, it can cause nerve damage [7]. Therefore, in the face of old fractures, we should choose reasonable surgical methods to avoid further compression of the fractured vertebrae, aggravating kyphosis, and damaging the spinal nerves.

In the early stage, Genant et al. proposed the Genant semi-quantitative method for fractures with different symptoms [8]. Smail et al. proposed the EVOSG classification and divided the OVCF into three types: wedge type, double concave type and collapse typ, but there is no classification of nerve damage [9]. Subsequently, Heini et al. [10] further improved and proposed the Heini classification according to clinical and imaging findings, but there was crosses overlap between different types, which was not conducive to clear clinical guidance. In addition, this classification does not reflect the severity and characteristics of osteoporotic fractures. In 2013, AO [11] proposed improved AO classification on the basis of the original, and comprehensively evaluated fracture classification from three aspects: morphological classification, neurological status and clinical correction index. However, this classification system is mainly applicable to high-energy fractures, not suitable for OVCF characterized by low-energy damage. Therefore, so far, there is still no appropriate classification and corresponding treatment scheme for the old OVCF.

Herein, in this study, we retrospectively analyzed 238 cases of patients with old OVCF admitted to our hospital from 2013 to 2016. According to the imaging morphological changes and clinical manifestations, the above patients were graded, and the treatment plan for each type was formulated. Meanwhile, VAS score, ODI score, ASIA grading and imaging results were used to evaluate the efficacy of corresponding treatment regiments.

## Methods

### Patients

In total, we retrospectively analyzed 238 OVCF patients admitted to our hospital from June 2013 to 2016. Among them, 110 cases were male and 128 cases were female. The average age of the included patients was 63.1 ± 6.7, ranging from 52 to 92 years. The inclusion criteria were: (1) A history of minor trauma; (2) Have a clear back pain; (3) Bone mineral density (BMD): T score ≤ -2.5; (4) OVCF clinical manifestations consistent with imaging findings; (5) Fracture to hospital interval ≥ 8 weeks; The exclusion criteria were: (1) Pathological vertebral fracture caused by tumor; (2) Abnormal coagulation function and poor cardiopulmonary function cannot tolerate surgery; (3) Surgery is not available for other reasons; (4) Unable to adhere to follow-up. This study was approved by our hospital. Written informed consent was obtained from each patient and the study was conducted in accordance with the Declaration of Helsinki.

According to clinical manifestations and imaging examination, the patients were divided into different grades. I: The patient had low back pain, X-ray showed wedge deformation of the vertebral body, and no signs of instability were seen on the dynamic radiograph. CT showed the cavity inside the vertebral body and MRI showed abnormal signals in the cavity inside the vertebral body. Specifically, when the cavity is liquid, T1WI is converted into low signal and T2WI is high signal. When there is gas in the cavity, T1WI and T2WI are both low signals. When there is gas and liquid mixing, T1 and T2 are both mixed signals. II: The patient had low back pain. X-ray showed wedge shape of the vertebral body. Dynamic radiograph showed instability of the vertebral body. In addition, CT showed vertebral cavity and MRI showed abnormal signal in vertebral body. III: The patient has low back pain, accompanied by lower limb pain, numbness, weakness, or intermittent claudication. X-ray showed wedge deformation of the vertebral body, and CT showed that the spinal canal occupied space and spinal canal stenosis. MRI showed compression of the dural sac and spinal stenosis at the same level. IV: The patient has low back pain, which seriously affects life, with obvious kyphosis in the back. X-ray showed severe loss of height of the injured vertebrae, kyphosis of the spine. CT showed cavities in the vertebral body, and MRI showed abnormal signals in the vertebral body. V: The patient's discomfort and imaging findings were a mixture of the above categories. The grading is independently assessed by two experienced doctors. If the results are consistent, they will be adopted. If the results are inconsistent, the third doctor will be invited to participate in the assessment, and the evaluation results will be decided by majority opinion.

### Surgical techniques

According to the above grading, different treatment schemes are adopted, as shown in Table 1. Vertebral augmentation: the injured vertebra was positioned percutaneous. A channel was inserted from the pedicle and bone cement was injected (Percutaneous vertebroplasty, PVP). In the presence of kyphosis, an airbag can be inserted before the cement is injected to expand and reduce the vertebral body (Percutaneous kyphoplasty, PKP). Posterior reduction fixation and fusion: posterior approach exposes the injured vertebrae and posterior structures of upper and lower vertebrae. The injured vertebra was inserted with short screws through the pedicle, while the upper and lower vertebrae were inserted with cement-enhanced screws through the pedicle and the injured vertebra was placed, or the injured vertebra could be put without screws. After reduction, the pedicle was inserted into the channel and bone cement was injected for reinforcement. The posterior lamina was bristled and the facet joints are destroyed after bone grafting. Posterior decompression and reduction fixation and fusion: posterior approach exposes injured vertebrae and posterior structures of upper and lower vertebrae. Shorter screws were placed through the pedicle of the injured vertebrae, and cement-enhanced screws were placed through the pedicle of the upper and lower vertebra. The posterior laminectomy of the injured vertebra was performed, and then the injured vertebra was reduced with a screw rod. The residual laminectomy was performed after the bruising, and the facet joints were destroyed and bone grafting was performed. Posterior osteotomy and fusion internal fixation: posterior approach exposes injured vertebrae and posterior structures of upper and lower vertebrae. The injured vertebrae can choose to insert shorter screws or no screws according to the osteotomy method, and the upper and lower vertebrae are inserted through the pedicle of bone cement strengthening screws. According to the degree of wedge deformation of the injured vertebrae and the degree of kyphosis, choose Ponte, SPO, PSO, VCR and other osteotomy correction methods, and then use the screw rod to restore the injured vertebra. The residual laminectomy was performed after the bruising, and the facet joints were destroyed and bone grafting was performed. In patients with kyphosis and mixed kyphosis requiring orthopedics, bone cement nail reinforcement is recommended. If the instability type requires screw fixation or T score ≤ -3.0, it is also recommended to use bone cement nail reinforcement.

**Table 1:** The grading criteria of osteoporotic vertebral compression fractures patients and the corresponding surgical procedures

Grade	Grading criterias	Surgical methods
Grade I (N = 86)	1. Recalcitrant back pain, increased pain after activity 2. X-ray showed wedge-shaped changes in the vertebral body, and some patients could see the injured vertebral cavity; CT showed a wedge-shaped change with an internal cavity; MRI showed that the height of the injured vertebra was lost and the internal signal of the cavity was abnormal	Vertebral augmentation
Grade II (N = 60)	1. Recalcitrant back pain, increased pain after activity 2. X-ray showed wedge-shaped changes in the injured vertebra, while dynamical X-ray film showed obvious changes in the angle of upper and lower endplates and local instability; CT showed a wedge-shaped changes with an internal cavity; MRI showed that the height of the injured vertebra was lost and the internal signal of the cavity was abnormal	Posterior reduction fusion internal fixation or combined vertebral augmentation
Grade III (N = 44)	1. Recalcitrant back pain, increased pain after activity with radiating pain in the lower extremities or intermittent claudication 2. X-ray showed loss of height of injured vertebra; CT showed wedge-shaped lesions with internal cavities, lumbar spinal stenosis, and compression of the dural sac; MRI showed that the height of the injured vertebra was lost, the signal was abnormal, the dural sac was compressed, and the same level of spinal canal stenosis	Posterior decompression and reduction fusion and internal fixation
Grade IV (N = 30)	1. Recalcitrant back pain, increased pain after standing for a long time 2. X-ray showed a wedge-shaped change with severe height loss and kyphosis of the spine; CT showed wedge-shaped changes of injured vertebra, internal cavity and kyphosis deformity of spine; MRI showed loss of injured vertebral height, kyphosis of the spine, abnormal signals inside the injured vertebra	Posterior osteotomy with internal fixation and fusion
Grade V (N = 18)	1. Recalcitrant back pain, aggravated after activity; Or accompanied by radiating pain in lower limbs, intermittent claudication; 2. X-ray showed a wedge-shaped change and kyphosis of the spine; CT showed wedge-shaped change of injured vertebra, kyphosis deformity of spine, lumbar spinal canal stenosis and dural sac compression; MRI showed that the height of the injured vertebra was lost, the signal was abnormal, the dural sac was compressed, and the same level of spinal canal stenosis	Posterior osteotomy (decompression) with internal fixation and fusion

The grading is independently assessed by two experienced doctors. If the results are consistent, they will be adopted. If the results are inconsistent, the third doctor will be invited to participate in the assessment, and the evaluation results will be decided by majority opinion

### Postoperative treatment

In cases of uneventful surgeries, all patients were required to be confined to bed for about 3 days after surgery. The patients were allowed appropriate ambulation 3 days after surgery while wearing a custom-made thoracolumbar orthosis. Subsequently, patients were encouraged to gradually increase the amount of exercise, perform lower back muscles strengthening exercises as soon as possible, and resume work depending on their speed of recovery. In addition, the patients were instructed to continue to receive regular anti-osteoporosis treatment in the outpatient clinic after discharge and to undergo regular reexamination.

### Evaluation methods and follow-up

Observe and record the visual analog score (VAS), oswestry disability index (ODI) score, kyphosis Cobb angle, vertebral sagittal index, nerve functional AISA rating. Clinical examination, X-ray films, CT and MRI were performed to assess fracture healing and vertebral height loss. Follow-up was conducted in the form of a questionnaire and the follow-up period was 12–38 months, with an average follow-up period of 18.5 months.

### Statistical analysis

SPSS 17.0 (IBM, New York, USA) was applied to analyze all data. Enumeration data were evaluated by χ^2^ test; Measurement data were evaluated by T-test. *P* < 0.05 was considered as statistical difference.

## Results

### Visual analog score (VAS)

Table 2 showed the changes of VAS preoperatively and postoperatively in 238 symptomatic old OVCF patients. In patients with grade I, the preoperative VAS was 8.00 ± 0.69, and the VAS were 2.20 ± 0.61 and 2.12 ± 0.74 at 12 months postoperatively and the last follow-up. There were significant differences in the VAS at 12 months postoperatively and the last follow-up as compared with the preoperative VAS (*P* < 0.05). In patients with grade II, VAS were 72.82 ± 7.78, 85.17 ± 5.26 and 84.17 ± 5.30 respectively at preoperation, postoperation 12 month and the last follow-up. It is worth noting that compared with preoperative VAS, postoperative VAS was significantly reduced after treatment with posterior reduction fusion internal fixation or combined vertebral augmentation (*P* < 0.05). In patients with grade III, the VAS at preoperation, postoperation 12 months and the last follow-up were 75.00 ± 6.66, 87.00 ± 5.01 and 85.07 ± 4.43. After treatment with the posterior decompression and reduction fusion and internal fixation, the VAS sharply decreased (*P* < 0.05). In patients with grade IV and V, VAS at 12 months postoperatively and at the last follow-up were 2.20 ± 0.58, 2.16 ± 0.84 and 2.20 ± 0.76, 2.11 ± 0.90, respectively. Similar to grade I-III, the postoperative VAS in grade IV and V were also significantly reduced (*P* < 0.05).

**Table 2:** The comparison of VAS scores between pre-operation and final follow-up in 238 osteoporotic vertebral compression fractures patients

Time	Grade I (N = 86)	Grade II (N = 60)	Grade III (N = 44)	Grade IV (N = 30)	Grade V (N = 18)
Preoperation	8.00 ± 0.69	8.05 ± 0.75	8.14 ± 0.82	8.00 ± 0.74	8.11 ± 0.76
Postoperation 12 m	2.20 ± 0.61^a^	2.11 ± 0.54^a^	2.21 ± 0.60^a^	2.20 ± 0.58^a^	2.16 ± 0.84^a^
Final follow-up	2.12 ± 0.74^a^	2.03 ± 0.78^a^	2.16 ± 0.78^a^	2.20 ± 0.76^a^	2.11 ± 0.90^a^

*VAS* visual analog score

^a^Compared with preoperative VAS, *P* < 0.05

### Oswestry disability index (ODI)

Table 3 showed the changes of ODI scores preoperatively and postoperatively in 238 symptomatic old OVCF patients. In patients with grade I, the preoperative ODI score was 69.63 ± 2.93, and the ODI scores were 44.25 ± 3.10 and 40.07 ± 2.65 at 12 months postoperatively and the last follow-up. There were significant differences in the ODI scores at 12 months postoperatively and the last follow-up as compared with the preoperative VAS after treatment with vertebral augmentation (*P* < 0.05). In patients with grade II, ODI scores were 70.23 ± 2.30, 41.25 ± 3.31 and 39.23 ± 2.56 respectively at preoperation, postoperation 12 months and the last follow-up. It is worth noting that compared with preoperative ODI score, postoperative ODI scores were significantly reduced (*P* < 0.05). In patients with grade III, the ODI scores at preoperation, postoperation 12 month and the last follow-up were 70.18 ± 1.87, 42.13 ± 2.27 and 40.09 ± 2.24. After treatment with the posterior decompression and reduction fusion and internal fixation, the ODI scores sharply decreased (P < 0.05). In patients with grade IV and V, ODI scores at 12 months postoperatively and at the last follow-up were 42.54 ± 2.01, 40.93 ± 1.90 and 39.07 ± 1.72 and 39.56 ± 2.33, respectively. Similar to grade I-III, the postoperative ODI scores in grade IV and V were also significantly reduced (*P* < 0.05).

**Table 3:** The comparison of ODI scores between pre-operation and final follow-up in 238 osteoporotic vertebral compression fractures patients

Time	Grade I (N = 86)	Grade II (N = 60)	Grade III (N = 44)	Grade IV (N = 30)	Grade V (N = 18)
Preoperation	69.63 ± 2.93	70.23 ± 2.30	70.18 ± 1.87	70.00 ± 2.23	70.56 ± 2.25
Postoperation 12 m	44.25 ± 3.10^a^	41.25 ± 3.31 ^a^	42.13 ± 2.27 ^a^	42.54 ± 2.01 ^a^	40.93 ± 1.90 ^a^
Final follow-up	40.07 ± 2.65 ^a^	39.23 ± 2.56 ^a^	40.09 ± 2.24 ^a^	39.07 ± 1.72 ^a^	39.56 ± 2.33 ^a^

^a^Compared with preoperative ODI, *P* < 0.05

*ODI* oswestry disability index

### Sagittal index (SI)

Table 4 showed the changes of SI preoperatively and postoperatively in 238 symptomatic old OVCF patients. As presented in Table 4, the SI of I-V grades before surgery were 89.78 ± 2.07, 72.82 ± 7.78, 75.00 ± 6.66, 71.83 ± 5.14 and 71.72 ± 6.64, respectively. At 12 months postoperatively, SI were 87.42 ± 5.08, 85.17 ± 5.26, 87.00 ± 5.01, 86.12 ± 4.09 and 85.28 ± 4.62 in I-V grades. In the last follow-up, the SI of I–V grades were 86.90 ± 6.28, 84.17 ± 5.30, 85.07 ± 4.43, 84.50 ± 4.67 and 83.39 ± 4.97, respectively. Importantly, SI increased significantly in patients of all grades after graded surgery compared with preoperative levels (*P* < 0.05).

**Table 4:** The comparison of SI between preoperation and final follow-up in 238 osteoporotic vertebral compression fractures patients

Time	Grade I (N = 86)	Grade II (N = 60)	Grade III (N = 44)	Grade IV (N = 30)	Grade V (N = 18)
Preoperation	89.78 ± 2.07	72.82 ± 7.78	75.00 ± 6.66	71.83 ± 5.14	71.72 ± 6.64
Postoperation 12 m	87.42 ± 5.08 ^a^	85.17 ± 5.26 ^a^	87.00 ± 5.01 ^a^	86.12 ± 4.09 ^a^	85.28 ± 4.62 ^a^
Final follow-up	86.90 ± 6.28 ^a^	84.17 ± 5.30 ^a^	85.07 ± 4.43 ^a^	84.50 ± 4.67 ^a^	83.39 ± 4.97 ^a^

*SI* sagittal index

^a^Compared with preoperative SI, *P* < 0.05

### Imaging assessment

**Fig. 1:**
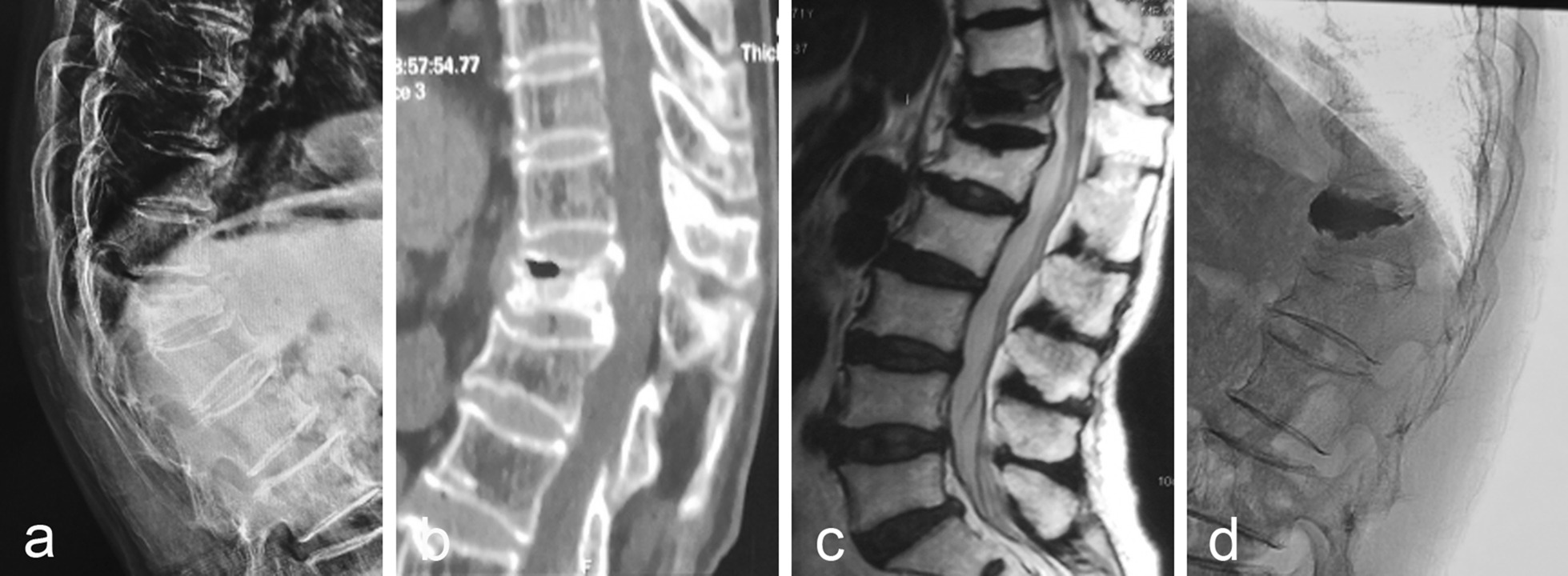
A 71-year-old female patient (Grade I) presented with minor trauma 5 months ago and low back pain 2 months ago, which was associated with activity. **a** The X-ray showed wedge-shaped changes of the T12 vertebra with kyphosis of the spine; **b** CT showed anterior collapse of the T12 vertebra with low-density shadow and peripheral sclerosis; **c** MRI T2 showed low signal in the vertebral body; **d** Postoperative X-ray showed that the height of the injured vertebra recovered well and the kyphosis deformity was corrected

There are 86 cases of patients with grade I. During the follow-up period, a total of 7 patients showed different degrees of vertebral height loss, and the SI decreased from 90.00 ± 2.34 to 71.67 ± 1.72. MRI examination showed no obvious abnormal signal, so it was not treated. Figure 1 showed the imaging data of one patient in grade I before and after treatment with vertebral augmentation. The imaging results depicted that after treatment with vertebral augmentation, the height of the injured vertebra of patient recovered well and the kyphosis deformity was corrected.

**Fig. 2:**
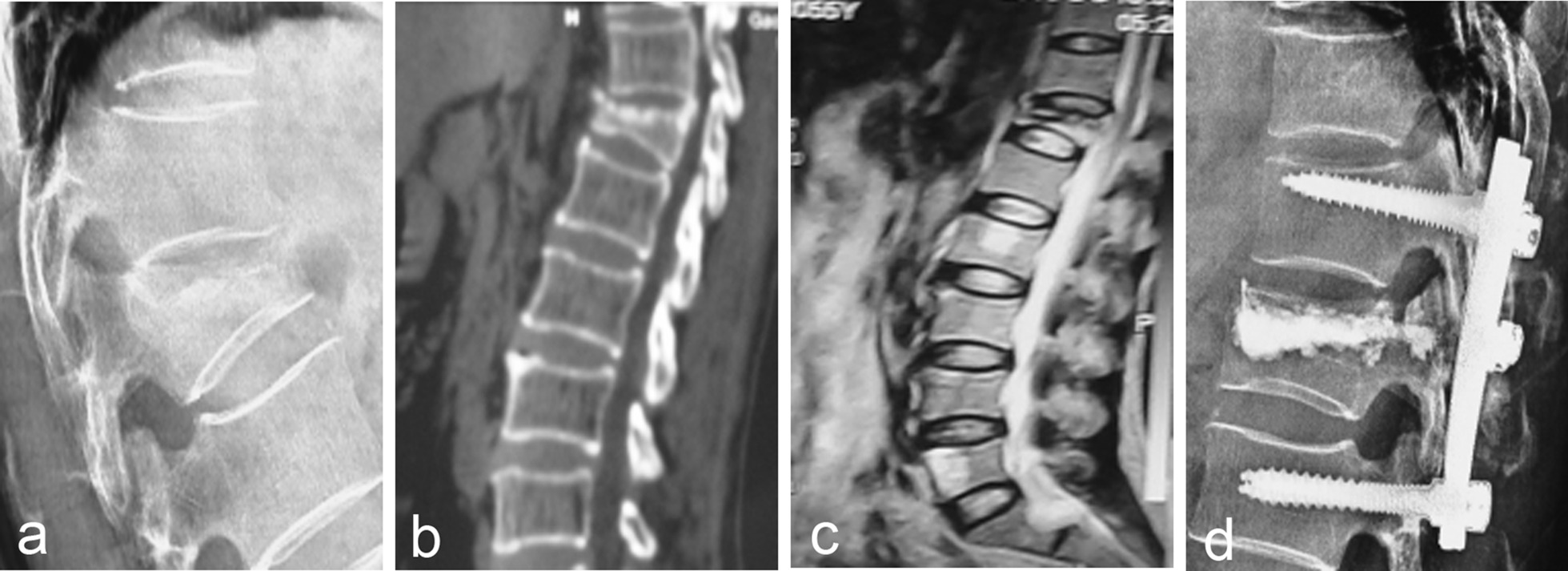
A 56-year-old female patient (Grade II) presented with low back pain due to heavy lifting 7 months ago and aggravated low back pain 2 months ago, which was correlated with activity. **a** The X-ray showed wedge-shaped changes in the T12 vertebra; **b** CT showed wedge-shaped changes in the vertebral body, collapse of the upper endplate of the T12 vertebral body, unhealed vertebral fracture, and small fractures in front of the injured vertebral body; **c** MRI T12 showed low signal in the vertebral body; **d** Postoperative X-ray showed that the patient underwent posterior reduction fixation and fusion and vertebral augmentation

There are 60 cases of patients with grade II. Among them, 1 case showed height loss of vertebral body; No obvious abnormality was found in MRI re-examination, so no treatment was performed, but continued observation is required. Figure 2 showed the imaging data of one patient in grade II before and after treatment with posterior reduction fixation and fusion and vertebral augmentation. Preoperative imaging data showed wedge shaped changes in the T12 vertebral body, small fractures in front of the injured vertebral body and low signals in the vertebral body. Postoperative X-ray showed that the patient underwent posterior reduction fixation and fusion and vertebral augmentation, and the patient recovered well.

**Fig. 3:**
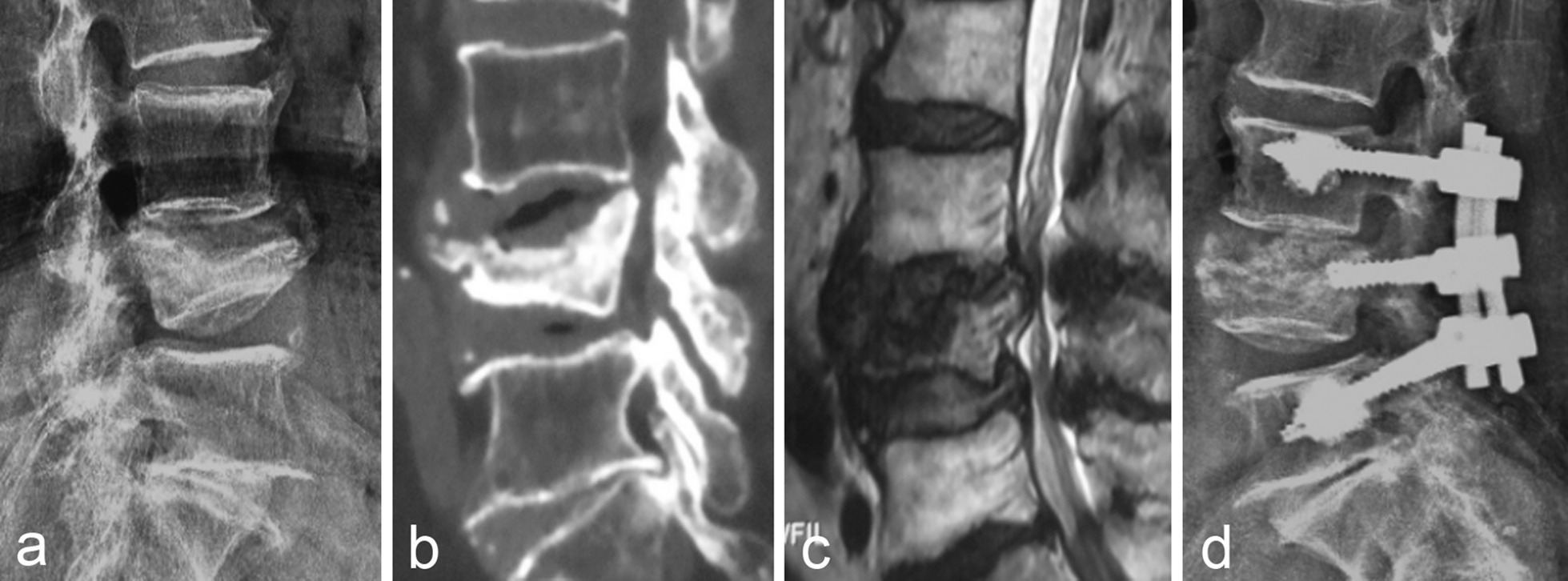
A 73-year-old female patient (Grade III) presented with mild back pain due to trauma 4 months ago, and presented with increased pain and numbness and weakness in both lower limbs 1 month ago, accompanied by intermittent claudication. **a** Lumbar spine X-ray showed the height of the L4 vertebra was lost and the endplate collapsed; **b** Sagittal CT showed collapse of the L4 vertebral body, low-density shadow in the vertebral space, protrusion of fracture block into the spinal canal, and spinal stenosis at the same level; **c** MRI T2 showed abnormally low signal in the L4 vertebra, with obvious dural compression and spinal stenosis at the same level; **d** Postoperative X-ray showed that the patient underwent posterior decompression and bone graft fusion with cement-reinforced internal fixation

There were 44 patients with grade III disease, and there was no height loss and local kyphosis. All patients with nerve injury showed improvement after surgery. The ASIA grade was improved from 9 cases of grade C and 1 case of grade D to the postoperative grade E. Figure 3 showed the imaging data of one patient in grade III before and after treatment with posterior decompression and reduction fusion and internal fixation. After treatment with posterior decompression and bone graft fusion with cement-reinforced internal fixation, the height of the injured vertebra of patient recovered well and spinal stenosis was improved.

**Fig. 4:**
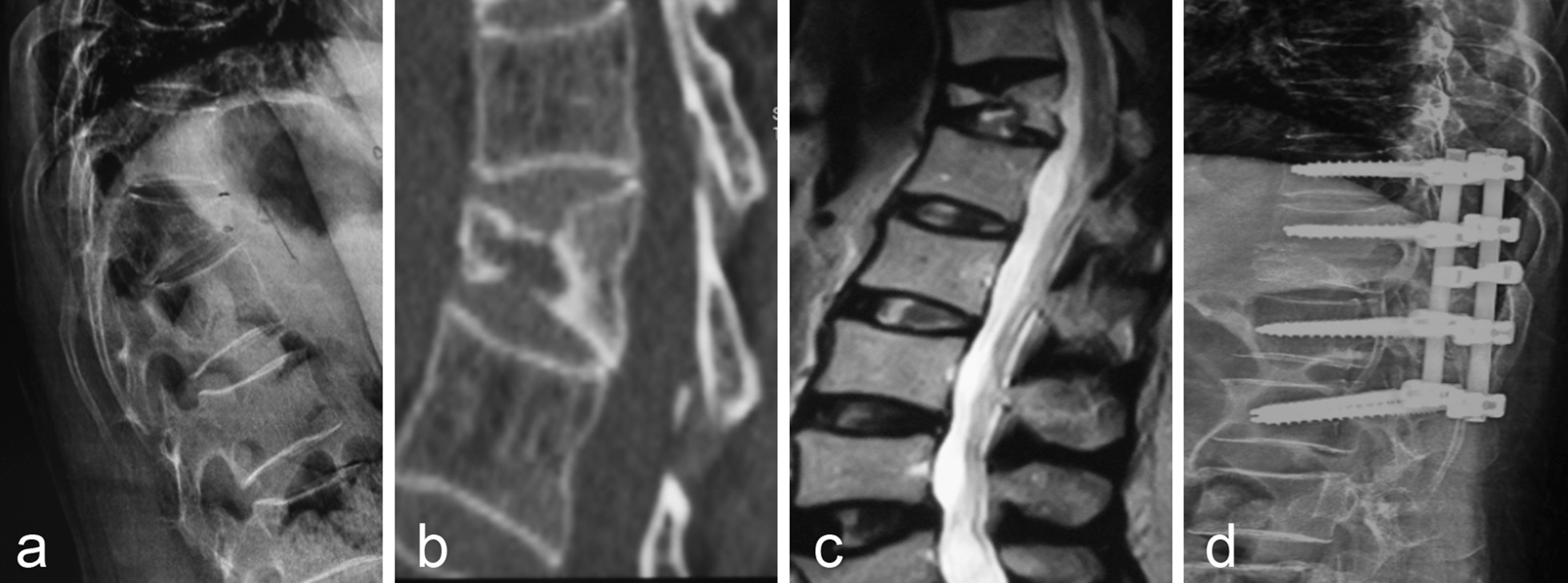
A 63-year-old female patient (Grade IV) presented with minor trauma 10 months ago and low back pain 2 months ago, which was associated with activity. **a** Thoracic X-ray showed a height loss of the T12 vertebral body and kyphosis of the thoracolumbar segment; **c** MRI T2 image showed low signal in the T12 vertebra; **d** Postoperative X-ray showed that the patient underwent posterior osteotomy and fusion internal fixation

There were 30 patients with grade IV. During the follow-up period, there were 2 cases of vertebral height loss, and the SI index decreased from 86.0 ± 2.0 to 71.5 ± 1.5. The patient developed severe back pain with local kyphosis angle of 25.5° ± 3.54°. Figure 4 showed the imaging data of one patient in grade IV before and after treatment with posterior decompression and reduction fusion and internal fixation. After revision surgery, the patient's discomfort symptoms were relieved.

**Fig. 5:**
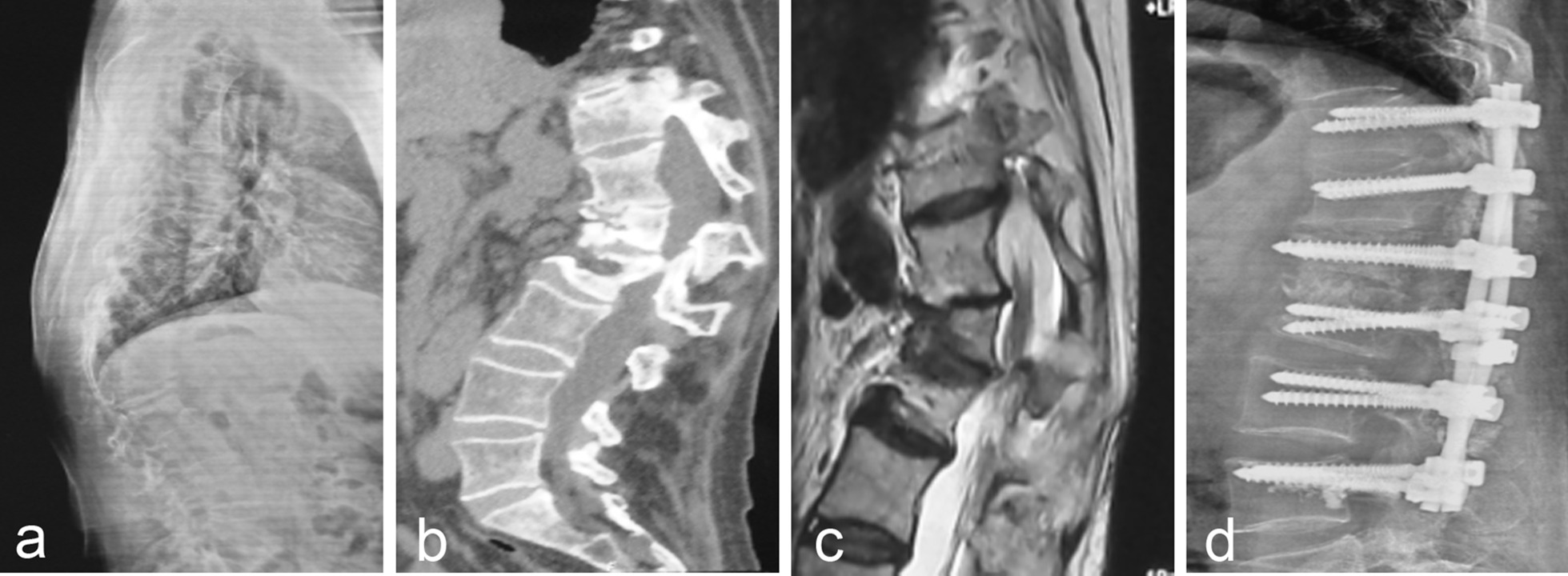
A 55-year-old female patient (Grade V) presented with back pain due to a fall 6 months ago and increased back pain accompanied by numbness and weakness in both lower limbs 2 months ago. **a** Thoracic X-ray showed wedge-shaped changes of L1 and L2 vertebra with kyphosis of thoracolumbar segment; **b** CT showed wedge-shaped changes in L1 and L2 vertebrae, kyphosis of the spine, and lumbar spinal stenosis; **c** Sagittal MRI T2 showed low signal of L1 and L2 vertebrae, kyphosis of thoracolumbar segment, spinal canal stenosis and obvious compression of dural sac; **d** Postoperative X-ray showed that the patient underwent posterior osteotomy and decompression and reduction combined with internal fixation

There were 18 patients with grade V. During the follow-up, 1 patient had a loss of vertebral height, but the patient was not treated because of no discomfort. Among the patients with nerve injury, the AISA grade of 6 patients with nerve injury was improved from grade C (5 cases), grade D (1 case) to grade D (4 cases) and grade E (2 cases). Figure 5 showed the imaging data of one patient in grade V before and after treatment with posterior osteotomy (decompression) with internal fixation and fusion.

## Discussion

OVCF is a common and frequently-occurring disease in the elderly. For acute fresh fractures, patients tend to be treated with minimally invasive vertebral augmentation, and studies have confirmed that satisfactory results can be achieved [12]. For the treatment of old OVCF, there is no international consensus. As mentioned earlier, many scholars have proposed classification for old OVCF, but there are many problems as follows: (1) The classification lacks clinical manifestations such as nerve damage; (2) There is no corresponding treatment plan for typing; (3) There is a duplication between the types, which does not guide clinical treatment well; (4) There is no specificity in the classification, which also contains high energy injury fractures. Therefore, based on the previous research, our study proposes five new types of grades, and proposed corresponding treatment plans for the grades.

Vertebral augmentation (VA) mainly includes percutaneous vertebroplasty (PVP) and percutaneous kyphoplasty (PKP) [13]. In meta-analysis in 2006, 19 studies on the treatment of OVCFs by PVP were included, involving a total of 2086 patients. The results showed that the postoperative VAS of the patients was significantly improved, and the incidence of surgical complications was less than 1% [14]. Another meta-analysis published at the same time included 1710 VCFs patients who had received PKP, and the postoperative VAS of the patients was significantly reduced, and the vertebral height and kyphosis were also improved [15]. In 2016, Clark's [16] prospective randomized controlled study showed that the pain relief in the surgery group was more obvious than that in the conservative treatment group, with a statistically significant difference. In this study, the cavities in the vertebral body were visible in the imaging examination for the patients of grade I. The local instability of the fractured vertebral body resulted in intractable low back pain. For the reasons of symptoms, vertebral augmentation was performed according to the literature recommendation [17]. To our satisfaction, the postoperative pain was significantly relieved.

The key destination of posterior reduction fusion internal fixation was to fix unstable segments. The advantages of starting from the posterior approach are less bleeding, less trauma, and simple access. At the same time, the front, middle and back are fixed at three ends. It has excellent mechanical properties, realizes the effect of three-dimensional fixation, and can often achieve anatomical reduction in cases that are difficult to reduce [18]. In this study, the dynamic position film of the patients with grade II showed obvious pseudo-joint movement, and the pain was related to the change of movement or body position. Therefore, this type of patient undergoes posterior reduction fusion internal fixation, and if necessary, combined with vertebral augmentation, to achieve the purpose of stabilizing the spine and alleviating the pain caused by height loss and local instability. Finally, the joint was fixed and fused with the nail rod to eliminate the movement of the prosthesis. In this study, the SI of the injured vertebra of 60 patients was improved from 72.82 ± 7.78 to 84.17 ± 5.30, the height of the injured vertebra was recovered satisfactorily, and the stability of the spine was well reconstructed, with an exact effect.

Posterior decompression and reduction fusion and internal fixation is based on reduction fixation for decompression and the most common approach is from the back to the spine. During the procedure, the surgeon makes a longitudinal incision in the back, first connecting the screw and/or bone hook to the vertebral body, and then attaching the rod to the screw and/or bone hook. Finally, the bone graft is placed on the spine after orthopedic fixation to ensure postoperative fusion of the orthopedic site. Grade III patients were accompanied by nerve damage, and imaging showed spinal canal stenosis. The purpose of surgery was to relieve nerve compression and restore spinal stability. Previous, Park et al. depicted that this surgery was effective in relieving delayed neurologic compromises [19]. Besides, Lee et al. had shown that posterior decompression and reduction fusion and internal fixation can relieve spinal cord compression and nerve compression [20]. In our study, the postoperative follow-up patients showed significant relief of lower back pain, and the 10 patients with significant nerve injury showed significant improvement in postoperative AISA grading, which was consistent with the previously reported results.

Patients with grade IV had local instability of the vertebral body, and the biomechanics of the spine was destroyed [20, 21]. Later, the secondary collapse caused kyphosis, accompanied by low back pain, and even serious nerve damage [22]. For patients with this grade, the goal of surgery was to correct kyphosis and restore sagittal balance. In our study, we performed posterior osteotomy with internal fixation and fusion, and the results were consistent with that reported by [23], which was satisfactory. However, Uchida et al. advocated the anterior orthopedic surgical strategy [24] and believed that the anterior medial column was the key site of spinal deformity in patients with old fractures, and the anterior surgical approach could achieve the purpose of decompression and orthopedic more directly and thoroughly. For these different viewpoints, we need to pay attention to the fact that anterior surgery is more traumatic and more complicated than posterior surgery. Currently, posterior surgery is mostly used. For the surgeon, the posterior anatomy is more familiar, the surgical trauma is less, and simple posterior surgery can also achieve similar effects to the combined approach [23]. The conditions of grade V patients were more complicated, with mixed symptoms. Therefore, the method of treatment should be determined based on the main symptoms. In this study, 18 patients received posterior surgery, including 12 patients with severe kyphosis who underwent posterior osteotomy and fusion and internal fixation, and 6 patients with severe spinal stenosis who underwent posterior decompression and internal fixation, all of which achieved satisfactory results.

Since most patients are associated with severe osteoporosis, postoperative complications such as screw loosening and vertebral height loss may occur. Therefore, anti-osteoporosis treatment is particularly important in the treatment of old OVCF. Studies have shown that the technique of bone cement augmentation can significantly improve the pull-out resistance of the screw [25]. Therefore, according to the bone condition of patients, some patients were augmented with bone cement. From the follow-up findings, the surgical method has achieved remarkable results. However, there were still patients with postoperative height loss of injured vertebra, which was speculated to be related to the patient's failure to strictly follow the doctor's advice on anti-osteoporosis treatment.

There are some limits in our study. First, there are fewer cases included in the study and the follow-up time was not long enough. In addition, the long-term efficacy of the surgery needs to be verified with longer follow-up. Second, this study is a single-center study, and the experience of the surgeon and personal preference may cause differences in results.

## Conclusion

Patients with OVCF are generally elderly and most of them have medical diseases, which increases the difficulty of treatment. Through graded surgery, the injured vertebrae can be effectively restored, local instability can be eliminated, nerve compression can be relieved, and kyphosis deformed can be corrected to achieve the purpose of reconstructing the stability of the spine. The quality of life of the patient is significantly improved, and the final therapeutic effect is satisfactory.

## Data Availability

The datasets used and/or analysed during the current study available from the corresponding author on reasonable request.

## Abbreviations

OVCF: Osteoporotic vertebral compression fractures
VAS: Visual analog score
ODI: Oswestry disability index
SI: Sagittal index
